# Exploring the definitions of gender bias in healthcare literature: a scoping review protocol

**DOI:** 10.1016/j.mex.2025.103545

**Published:** 2025-08-05

**Authors:** Silvia Bargeri, Laura A. Schaap, Greta Castellini, Silvia Gianola, Tiziano Innocenti, Raymond Ostelo, Rossella Tomaiuolo, Andres Vidal-Itriago, Sidney Rubinstein

**Affiliations:** a Unit of Clinical Epidemiology, IRCCS Istituto Ortopedico Galeazzi, Milan, Italy; b Department of Health Sciences, Faculty of Science, Vrije Universiteit Amsterdam, Amsterdam, The Netherlands; c Amsterdam Public Health Research Institute, The Netherlands; d Amsterdam Movement Sciences, Vrije Universiteit Amsterdam, The Netherlands; e GIMBE Foundation, Bologna, Italy; f Department of Epidemiology and Data Science, Amsterdam UMC, Vrije Universiteit & Amsterdam Movement Sciences, Musculoskeletal Health, Amsterdam, The Netherlands; g IRCCS Istituto Ortopedico Galeazzi, Milan, Italy; h Faculty of Medicine, Vita-Salute San Raffaele University, Milan, Italy; i Medical Library, Vrije Universiteit Amsterdam, Amsterdam, The Netherlands

**Keywords:** Gender bias, Gender inequities, Gender stereotypes, Scoping review, Definitions

## Abstract

**Introduction:**

Despite growing recognition of gender bias in healthcare, there is a lack of clarity on how gender bias is defined and understood in the literature. Existing definitions often did not consider modern gender constructs, limiting their applicability to current healthcare practices. In order to address these issues, we aim to perform a scoping review (ScR) to systematically explore what definitions and which characteristics of gender bias are found in the literature.

**Methods:**

This protocol follows the Joanna Briggs Institute methodology for ScR and reported according to PRISMA-ScR guidelines. A comprehensive search strategy will be performed in MEDLINE, EMBASE, Scopus and other relevant sources (date of last search: January, 15th 2025). We will include any studies that aim to provide structured definitions or conceptual frameworks to define and/or understand gender bias within healthcare clinical settings. Data will be charted using a standardized form to capture the characteristics of definitions, along with the healthcare population and contexts where gender bias has been described. Definitions will be described assessing their relevance from an analytical perspective and their potential for implementation. The findings will be summarized descriptively, with summary tables and figures to highlight key characteristics of the included studies.

## Highlights

• Gender bias lacks a clear definition and understanding in healthcare literature.
• We will assess how gender bias is defined and its related characteristics.
• Foundation for future studies to help assess the impact of gender bias in practice.

## Graphical abstract

**Figure:**
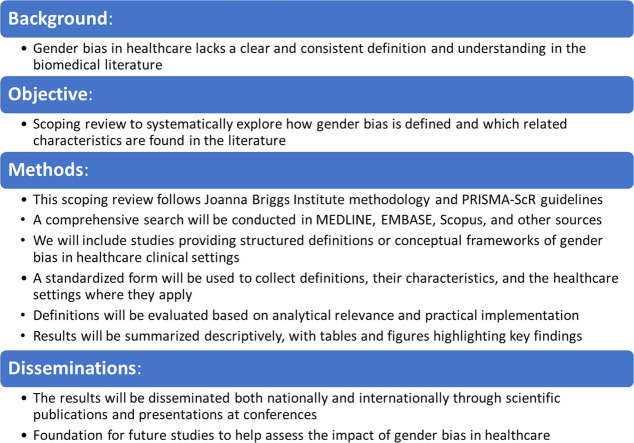

## Specifications table

**Table:** 

Subject Area	Medicine and Dentistry
More Specific Subject Area	Medicine, methods of synthesis of knowledge, study designs
Name of Protocol	Exploring the definitions of gender bias in healthcare literature: a scoping review protocol
Reagents/Tools	Not applicable
Experimental Design	Not applicable. This protocol describes a scoping review
Trial Registration	Not applicable. This scoping review is registered in the Open Science Framework (https://doi.org/10.17605/OSF.IO/QXRWH)
Ethics	This study does not involve human beings or non-human animals because it is secondary research
Value of the Protocol	• First scoping review of gender bias definitions in healthcare for an up-to-date, comprehensive and applicable understanding of gender bias. • Establish a framework for future studies to help assess the impact of gender bias in healthcare.

## Background

According to the World Health Organization, gender is a fundamental social determinant that shapes population health and contributes to health disparities [1]. Gender encompasses the socially assigned roles, expectations, and responsibilities attributed to individuals based on their gender expression and societal perceptions, distinct from biological sex, which refers to physiological, genetic, and hormonal characteristics [2].

While both sex and gender influence health, their mechanisms differ. Biological sex affects aspects like disease pathways (e.g., in relation to sex specific organs or hormones) or responses to medications (e.g., different pharmacodynamic), while gender shapes exposure to risks (e.g., different work exposure), health-related behaviors, and how people access or receive care (e.g., men that avoid help to appear strong or women expected to prioritize others) [3].

Healthcare systems are embedded in broader societal norms that frequently reproduce gender hierarchies [3,4]. These hierarchies tend to privilege what is socially coded as masculine, often to the detriment of women and gender-diverse individuals. As a result, the experiences of these populations are frequently undervalued, leading to systematic underdiagnosis, undertreatment, and exclusion from clinical research and decision-making processes [3,4]. A well-documented example is the case of women with cardiovascular diseases, who have historically been offered fewer diagnostic tests and medicines than men, resulting in poorer healthcare outcomes [5].

Traditionally, biomedical research has relied on male bodies as the default model, often treating women as "smaller men". This approach has led to significant knowledge gaps concerning sex- and gender-specific health responses and outcomes [6,7]. Furthermore, the continued failure to gather comprehensive demographic data, particularly data reflecting gender diversity, contributes to the exclusion and marginalization of not only of women but also of gender minorities [8,9].

This issue falls within the umbrella term of “gender bias”, which has commonly been defined as a favoritism toward one gender over another based on stereotyped beliefs about health, behavior, experiences, needs, and desires of men and women [10].

However, despite increasing attention to gender as a critical determinant of health, there is a lack of clarity on how gender bias is defined and understood within the biomedical literature. In this context, the European Commission has recently emphasized the importance of contemporary terminology and conceptual frameworks as a key strategy for detecting, monitoring, addressing, and ultimately reducing health disparities [11,12].

In fact, definitions published are likely to be outdated and to be heterogenous considering various perspectives, contexts, and methodologies [13,14]. Related terms to gender bias such as gender difference and inequality are often used interchangeably, contributing to conceptual confusion [15,16]. Gender difference refers to the observed differences displayed by females and males (or another gender) (e.g., gender difference in exposure to risks) [17], while gender inequality refers to the unequal treatment and opportunities experienced by individuals based on their gender [18]. Conflating gender differences with gender bias or inequality can obscure the structural and discriminatory mechanisms that drive health disparities.

Moreover, many existing definitions fail to include a comprehensive theoretical foundation, often relying on binary constructs which do not align with contemporary views on equality, diversity, and inclusion and do not capture the multifaceted intersectional social construct of gender [19].

It is now recognized that gender frequently interacts in an intersectional way with other social determinants of health, such as ethnicity, sexual orientation, socio-economic status and disability, increasing health disparities [20]. For example, lesbian, gay, bisexual, transgender, and queer plus (LGBTQ+) people with limited financial resources, may face multiple and overlapping forms of discrimination, resulting in poorer health outcomes [21].

To address these challenges, it is essential to develop clear, inclusive, and up-to-date definitions of gender bias in biomedical research. This would be the basis for future studies to evaluate how gender bias is perceived and operationalized in clinical practice, its potential impacts, and possibly to support the development of consensus-based frameworks and equity data collection practices. These practices should also account for ethical considerations around privacy, data security, and the potential misuse of sensitive information, in alignment with established European equality guidelines for data collection [11,12].

Therefore, our objective will be to systematically explore how gender bias is defined in the biomedical literature as a starting point to facilitate a more up-to-date, comprehensive, applicable and ethical understanding of gender bias in healthcare.

## Research questions

This scoping review will aim to explore gender bias definitions in the healthcare field. Specifically, the objective will be to answer the following primary research question:

• What definitions and which characteristics of gender bias are found in the literature?

## Description of protocol

### Study design

This scoping review protocol is reported according to the best practice guidance and reporting items for the development of scoping review protocols [22] and planned in accordance with the Joanna Briggs Institute methodology for scoping review [23]. The full scoping review will be reported to the Preferred Reporting Items for Systematic reviews and Meta Analyses extension for Scoping Reviews (PRISMA ScR) Checklist [24]. This scoping review is registered in the Open Science Framework (OSF) [25].

### Eligibility criteria

#### Study design

We will include any study design proving definitions or conceptual frameworks to define and/or understand gender bias, such as theoretical frameworks, literature review or commentaries. We will exclude studies that merely aim to describe or discuss examples of gender bias or national policies on gender equity in health, without proving structured definitions or conceptual frameworks.

#### Population and setting

We will include any population (e.g., musculoskeletal, orthopedics, cardiology) in any healthcare setting (e.g., inpatient or outpatient), level of care (e.g., primary, secondary care or tertiary care) or processes (i.e., diagnosis, treatment, or prognosis) in any country (e.g., high, middle, low income-countries).

We will exclude studies investigating gender bias in academic settings or in the workplace/careers of healthcare professionals, as well as studies related to the stigma caused by a specific health condition (e.g., HIV) or to specific population subgroups (e.g., widows, migrants, sex workers, drug users).

#### Outcomes

We will include studies that provide structured definitions or conceptual frameworks to define and/or understand the concept of gender bias, encompassing, but not limited to, gender inequities or inequalities, stereotypes, prejudices and discriminations. We will exclude studies assessing sex or gender differences in disease prevalence, risk factors, treatment outcomes without a specific focus on gender bias.

#### Language/date

No language or date restrictions will be applied.

More details about inclusion and exclusion criteria are reported in Appendix 1.

#### Information sources

##### Primary search approach

A medical information specialist (AV-I) will be consulted for constructing a comprehensive search strategy. The search strategy will undergo peer review by two senior information specialists, both with 20 years of experience, following the Peer Review of Electronic Search Strategies (PRESS) checklist [26].

In the first step, we will perform multiple pilots search in MEDLINE to retrieve relevant keywords and search terms for each aspect included in the search strategy. This will involve identifying terms in the titles and abstracts of articles considered relevant, focusing on those reporting definitions or conceptual frameworks in the results section (e.g., theoretical frameworks explicitly aimed at reporting definitions). In a second step, two reviewers (SB, LS) will iteratively pilot different search strings to develop a comprehensive search strategy, combining free-text terms and controlled vocabulary, where applicable. Decisions on whether to explode specific MeSH terms will be made with careful consideration of the term's relevance and the context of the research question, in consultation with the topic expert (SB).

The search strategy will be then used to search in Ovid MEDLINE, EMBASE and Scopus. The last search strategy developed (January 15th, 2025) is reported in Appendix 2A, with a sensitivity analysis using a different term in Appendix 2B No language, date or study design limits will be applied.

##### Additional methods

We will consider unpublished documents produced by any major international body or agency (e.g., WHO, European Institute for Gender Equality) (Appendix 3)**.**

#### Study selection of source of evidence

Given the anticipated large volume of records, we will use a multiple-step approach to perform the study screening:

Step 1. We will pilot a random sample of 5 % of the total identified records to estimate the prevalence of relevant articles that will be found in the main screening process. The prevalence of relevant articles will be calculated using the van Haastrecht’s formula [27,28]:

R=N*(r/(r+i))

Where:

• R: Estimated total number of relevant articles.
• N: Total number of records identified in the preliminary search.
• r: Number of relevant articles identified in the random sample.
• i: Number of irrelevant articles identified in the random sample.

This formula will help define the expected number of relevant articles and set an initial benchmark for subsequent screening.

Step 2. Once established the relevant articles from the random sample, two pairs of reviewers (SB, LS) will perform the main screening process, with one reviewer conducting the initial screening and the other performing a double-check of the assessment. Disagreement will be solved by discussion. ASReview [29], an Artificial Intelligence-aided screening tool that utilizes active learning to prioritize abstracts based on their predicted relevance [30,31], will be used.

To minimize biases inherent to AI in the selection and ranking of papers, we will follow a two-phase approach:

• 2.1: Active learning algorithms in ASReview [29] will reorder the abstracts based on their predicted relevance.
• 2.2: Screening will continue until a predefined stopping rule is satisfied, according to the SAFE procedure for active learning-based screening [32]:
  A. All key papers (identified during sampling and prior screenings) are marked as relevant.
  B. At least twice the estimated number of relevant records (R) are screened
  C. A minimum of 10 % of the total dataset has been screened.
  D. No relevant records have been identified in the last, for example, 100 records
• Step 3. To ensure that all relevant articles are captured, we will combine Step 2 with additional criteria. Specifically, we will review a sample of 100 records that may report definitions or frameworks in their introduction or methods section (e.g., primary studies that might use a definition but not actually define gender bias, such as interventional, observational or qualitative studies).

Finally, two reviewers (SB and LS) will independently assess the full texts of the papers included in the previous screening phase using Covidence [33], an online tool specifically designed to facilitate and streamline systematic review workflows. Discrepancies, if any, will be resolved through discussion or with the involvement of a third reviewer when needed and the number of conflicts will be reported in the final publication. Non-English full-text articles will be translated using DeepL [34], an advanced neural machine translation tool.

#### Charting the data

Two reviewers will extract data in Microsoft Excel using a data charting form that includes details such as authors, year, journal/source, study design, aim, definition of gender bias, attributes, terminology used, data sources, methods used to arrive at a definition, any patients/stakeholder involvement, healthcare context/setting applicable for the definitions, and the country in which the definition was developed (Table 1). The data charting forms will be piloted on 10 studies by two researchers for convenience (SB, LS). The researchers will review the data charting process (as it is an iterative process in scoping review) making any necessary revisions to the data charting form. Any disagreements will be resolved through discussion or with a third reviewer.

**Table 1:** Preliminary data extraction tool.

**General characteristics**

Citation details (e.g., author/s date, title, journal, volume, issue, pages):
Study design (e.g., consensus, narrative review, theory guide, commentary, mixed methods)
Number of authors involved
Research question or study purpose

**In which population and context has the gender bias been described?**

In which population has been assessed? (e.g., chronic pain, cardiology, orthopedics, musculoskeletal, oncology) In which healthcare setting? (e.g., in-patient; out-patient) In which level of care? (e.g., primary, secondary, tertiary care) In which healthcare process? (e.g., diagnosis, treatment, prognosis) In which country? (e.g., high, middle, low-income country)

**What definitions or theoretical frameworks of gender bias are found in the literature**?

How is gender bias defined?*
Which terminology is used? (e.g., gender differences/ gender inequities/ gender stereotypes/ gender prejudices/ gender discriminations)
What methods were used to arrive at a definition/framework? What type of data was included to arrive at a definition/framework? Were patients, stakeholders or experts included? How many? Which content expert were included? (e.g., gender medicine field, sociological field, any healthcare field)

⁎ We will report any definitions/theory found in included studies by assessing my themes emerged.

In addition to the descriptive synthesis, we will extract data adapting a previously published checklist on gender measures [19] to assess definitions’ relevance from an analytical perspective (e.g., inclusion, intersectionality) and their potential for implementation from a practical point of view (e.g., length or complexity of the definition, transferability). This checklist was developed in a previous scoping review [19] that systematically mapped gender-related measures, to describe the multiple demands in recent gender-sensitive research. It was designed as a reflection tool rather than a rigid quality assessment instrument, aiming to critically evaluate the conceptual and practical relevance of gender measures in epidemiology. In our scoping review, we will adapt it to assess definitions instead of measures, aligning it with our specific scope (Table 2).

**Table 2:** Adapted checklist to guide reflection on gender definitions [19].

**Reflection on gender definitions**

**Relevance from an analytical perspective**

Is the definition/framework inclusive? Does it rely on a non-binary understanding of gender? Does the definition/framework include the intersection or interaction of several social determinants of health? Is gender considered in relation with at least another social determinant of health (e.g., socio-economic status, ethnicity)? Is the definition/framework “symmetrical”, in the sense that participants of all genders are equally asked about their roles? Does the definition address different levels of gender processes, and within the levels, different dimensions? Or is it focused on one level, and/or one dimension?

**Potential for implementation from a practical point of view**

Is the definition/framework too long or complex? Is the terminology ambiguous or well explained? Has it been validated? Is the definition based on another published definition? Is it available in several countries? Or is it adaptable to different contexts? Is it transferable, or generalizable do other settings?

#### Collating, summarising and reporting the results

The findings will be summarized descriptively, integrating narrative text with summary tables and figures to illustrate key characteristics of the included studies. This approach will enable the creation of a comprehensive assessment of how gender bias is defined and understood within health literature.

Specifically, we will examine the variety of definitions and theoretical frameworks related to gender bias, along with the healthcare contexts and processes where gender bias has been described or assessed. The findings will include a synthesis of gender bias definitions that incorporate intersectional perspectives, emphasizing their practical relevance and potential for implementation. The implications of the definitions in terms of practical applications will be discussed. We will also highlight any gaps in the existing literature.

#### Consultation

Consultation with experts is recommended throughout the scoping review process [24]. The research team already includes content experts in gender medicine (RT, SB, SG). The team is balanced in terms of sex and gender, career background and progress, expertise in different fields (gender medicine, musculoskeletal, methodology) and nationality (Italian, Dutch) to ensure a full coverage assessment of the topic.

#### Disseminations

This project will be the first comprehensive systematic examination of gender bias definitions within healthcare. The results will be disseminated both nationally and internationally through scientific publications and presentations at conferences and seminars dedicated to the topic, as well as in social media platforms (such as Facebook, LinkedIn). By providing clearer conceptual and theoretical perspectives, this research aims to deepen the understanding of gender bias definitions in healthcare, raising awareness and expanding knowledge on gender bias. Ultimately, it could establish a foundation for future studies, such as expert consensus, to develop a unified definition of gender bias applicable across various research fields to help assess the impact of gender bias in healthcare.

### Limitations

While this scoping review aims to provide a comprehensive overview of how gender bias is defined in healthcare literature, some limitations must be acknowledged. First, due to the very large volume and heterogeneity of the literature in this area, we decided to focus this scoping review on mapping the existing definitions and theoretical frameworks as a necessary foundation for future research. Second, the diversity of definitions and conceptual frameworks across the healthcare field may make it challenging to standardize findings, and some relevant literature may be overlooked due to publication bias or inconsistencies in terminology. To address this, we developed search queries that balance sensitivity and specificity, aiming to ensure both relevance and inclusivity without compromising search quality.

Third, the use of ASReview software in screening should take into account possible biases in selection and ranking. To reduce this risk, we will implement several safeguards, including manually screening additional 100 records and consulting grey literature (e.g., WHO, EIGE). Moreover, it has been reported that, although human reviewers are considered the “gold standard,” they are not perfect (average error rate due to false inclusion/exclusion of 10.76 %, varying by topic) [35]. Therefore, automation tools with comparable accuracy may be acceptable when used alongside a human reviewer, offering potential savings in time and resources. Specifically, the application of a stopping rule will facilitate the screening of a substantially larger volume of records, which would otherwise be unmanageable through manual review alone.

Lastly, as a scoping review, this study will not critically appraise the quality of included studies, but rather aims to map the existing knowledge and describe how to guide reflection on gender definitions. Despite these challenges, our work represents a crucial step toward establishing a clearer and more applicable understanding of gender bias in healthcare.

## CRediT authorship contribution statement

**Silvia Bargeri:** Conceptualization, Methodology, Data curation, Investigation, Writing – original draft, Writing – review & editing. **Laura A. Schaap:** Conceptualization, Methodology, Data curation, Investigation, Supervision, Writing – review & editing. **Greta Castellini:** Conceptualization, Methodology, Data curation, Writing – review & editing. **Silvia Gianola:** Conceptualization, Methodology, Data curation, Writing – review & editing. **Tiziano Innocenti:** Conceptualization, Methodology, Data curation, Supervision, Writing – review & editing. **Raymond Ostelo:** Conceptualization, Methodology, Data curation, Supervision, Writing – review & editing. **Rossella Tomaiuolo:** Methodology, Validation, Data Curation, Writing – review & editing. **Andres Vidal-Itriago:** Methodology, Software, Data curation, Investigation, Writing – review & editing. **Sidney Rubinstein:** Conceptualization, Methodology, Data curation, Supervision, Writing – review & editing.

## Funding

SB, GC and SG are supported and funded by the Italian Ministry of Health - "Ricerca Corrente”.

## Ethics

Ethical approval will not be required for this scoping review as all data reviewed and collected will be obtained from publicly available sources.

## Patient and public involvement

No patients were involved in the protocol design.

## Declaration of interests

The authors declare that they have no known competing financial interests or personal relationships that could have appeared to influence the work reported in this paper.

## Figures and Tables

**Table:** 

**Inclusion criteria**	**Exclusion criteria**
**Study design**	
Any study design providing definitions, conceptual frameworks or conceptual models specifically aiming to define and/or understand the concept of gender bias such as, but not limited to, theoretical frameworks, literature reviews or commentaries.	Studies that merely aim to describe or discuss examples of gender bias, gender disparities, gender inequity or inequalities, gender differences or social determinants related to gender without offering a structured definition or conceptual framework to define and/or understand gender bias. Studies that primary examine national policies on gender equity in health without proving structured definitions, conceptual frameworks or conceptual models. Primary studies such as interventional, observational or qualitative studies, aiming to assess the impact of gender bias or perceptions of gender bias in clinical practice*
**Population and setting**	
Any population in any healthcare clinical practice setting (i.e., inpatient or outpatient), level of care (i.e., primary, secondary, tertiary care), or processes (diagnosis, treatment, or prognosis).	Academic settings, workplace setting, research settings, careers of healthcare professionals, as well as studies related to the stigma caused by a specific health condition (e.g., HIV) or to subcategory of population (e.g., widows, migrants, sex workers, drug users).
**Outcomes**	
Any definition, framework, concept or model specifically aiming to define and/or understand gender bias, encompassing, but not limited to, inequities, stereotypes, prejudices and discriminations related to gender.	Studies assessing sex or gender differences in disease prevalence, risk factors, treatment outcomes without a specific focus on gender bias.

⁎ Primary studies will be excluded but a sample of 100 studies will be checked for reporting relevant references (see Step 3 in Methods).

**Table:** 

**Search**	**Query**	**Results**
5	(1 or 2) and 3 and 4	**10,050**
4	exp "Intersectional Framework"/ OR exp "Social Theory"/ OR exp "Quality Assurance, Health Care"/ OR ("definit*" or "concept*" or "theor*" or "framework*" or "guideline*" or "polic*").ti,ab,kf.	3301,436
3	exp "Delivery of Health Care"/ or "Professional Practice"/ or exp "Health Care Sector"/ or exp "Right to Health"/ or "Health Policy"/ or "Patient Care"/ or exp "Health Care Quality, Access, and Evaluation"/ or exp "Universal Health Care"/ or ("healthcare" or (("medic*" or "clinic*" or "health*" or "patient*") adj2 ("care" or "service*" or "practice" or "access"))).ti,ab,kf.	10,005,335
2	(exp Health inequities/ or exp Healthcare Disparities/ or Prejudice/ or exp Diversity, Equity, Inclusion/) and (exp Gender identity/ or exp Sex/ or exp Gender role/ or (gender or sex or sexual).ti,ab,kf)	13,608
1	exp "Sexism"/ or exp "Gender Equity"/ or ("Sexism*" or (("Gender*" or "sex" or "sexual" or "man" or "men" or "Women" or "woman" or "non-binar*" or "male*" or "female*") adj3 ("inequ*" or "prejud*" or "discriminat*" or "bias*" or "stereotyp*" or "exclus*" or "disparit*" or "equit*" or "gap" or "inclus*" or "stigma*" or "parity" or "under-represent*" or "underrepresent*" or imbalanc*))).ti,ab,kf.	65,102

**Table:** 

**Search**	**Query**	**Results**
7	#5 NOT #6	**9128**
6	#5 AND ('conference abstract'/it OR 'conference paper'/it OR 'conference review'/it)	3271
5	(#1 OR #2) AND #3 AND #4	12,185
4	'intersectionality'/exp OR 'sociological theory'/exp OR 'definit*':ti,ab,kw OR 'concept*':ti,ab,kw OR 'theor*':ti,ab,kw OR 'framework*':ti,ab,kw OR 'guideline*':ti,ab,kw OR 'polic*':ti,ab,kw	3718,664
3	'health care delivery'/exp OR 'professional practice'/exp OR 'health care concepts'/exp OR 'right to health'/exp OR 'health care policy'/exp OR 'patient care'/exp OR 'health care quality'/exp OR 'universal health care'/exp OR 'healthcare':ti,ab,kw OR ((('medic*' OR 'clinic*' OR 'health*' OR 'patient*') NEAR/2 ('care' OR 'service*' OR 'practice' OR 'access')):ti,ab,kw)	13,484,252
2	('health disparity'/exp OR 'health care disparity'/exp OR 'prejudice'/exp OR 'diversity, equity and inclusion'/exp) AND ('gender identity'/exp OR 'sex'/exp OR 'sex role'/exp OR 'gender':ti,ab,kw OR 'sex':ti,ab,kw OR 'sexual':ti,ab,kw)	14,180
1	'sexism'/exp OR 'gender equity'/exp OR 'sexism*':ti,ab,kw OR ((('gender*' OR 'sex' OR 'sexual' OR 'man' OR 'men' OR 'women' OR 'woman' OR 'non-binar*' OR 'male*' OR 'female*') NEAR/3 ('inequ*' OR 'prejud*' OR 'discriminat*' OR 'bias*' OR 'stereotyp*' OR 'exclus*' OR 'disparit*' OR 'equit*' OR 'gap' OR 'inclus*' OR 'stigma*' OR 'parity' OR 'under-represent*' OR 'underrepresent*' OR 'imbalanc*')):ti,ab,kw)	89,471

**Table:** 

**Search**	**Query**	**Results**
5	(1 or 2) and 3 and 4	**10,042**
4	(TITLE-ABS-KEY ("definit*" OR "concept*" OR "theor*" OR "framework*" OR "guideline*" OR "polic*") OR AUTHKEY ("definit*" OR "concept*" OR "theor*" OR "framework*" OR "guideline*" OR "polic*"))	16,377,962
3	(TITLE-ABS-KEY ("healthcare" OR (("medic*" OR "clinic*" OR "health*" OR "patient*") W/2 ("care" OR "service*" OR "practice" OR "access"))) OR AUTHKEY ("healthcare" OR (("medic*" OR "clinic*" OR "health*" OR "patient*") W/2 ("care" OR "service*" OR "practice" OR "access"))))	4280,352
2	(TITLE-ABS-KEY ((("health" OR "healthcare") W/4 ("ineq*" OR "dispar*" OR "prejud*" OR "equity" OR "inclusion" OR "diversity")) AND (("gender*" OR "sex" OR "sexual") W/3 ("identit*" OR "role*" OR "orientation"))) OR AUTHKEY ((("health" OR "healthcare") W/4 ("ineq*" OR "dispar*" OR "prejud*" OR "equity" OR "inclusion" OR "diversity")) AND (("gender*" OR "sex" OR "sexual") W/3 ("identit*" OR "role*" OR "orientation"))))	4538
1	(TITLE-ABS-KEY ("sexism*" OR (("gender*" OR "sex" OR "sexual" OR "man" OR "men" OR "women" OR "woman" OR "non-binar*" OR "male*" OR "female*") W/3 ("inequ*" OR "prejud*" OR "discriminat*" OR "bias*" OR "stereotyp*" OR "exclus*" OR "disparit*" OR "equit*" OR "gap" OR "inclus*" OR "stigma*" OR "parity" OR "under-represent*" OR "underrepresent*" OR imbalanc*))) OR AUTHKEY ("sexism*" OR (("gender*" OR "sex" OR "sexual" OR "man" OR "men" OR "women" OR "woman" OR "non-binar*" OR "male*" OR "female*") W/3 ("inequ*" OR "prejud*" OR "discriminat*" OR "bias*" OR "stereotyp*" OR "exclus*" OR "disparit*" OR "equit*" OR "gap" OR "inclus*" OR "stigma*" OR "parity" OR "under-represent*" OR "underrepresent*" OR imbalanc*))))	190,255

**Table:** 

Database/Date of search	Results January 15th, 2025 without "nonbinar*"	Results June 17th, 2025 with "nonbinar*"	Results June 17th, 2025 "nonbinar*"	Results June 17th, 2025 "nonbinar*" only
**MEDLINE/20,250,115**	**10,050**	**10,726**	**10,721**	**5**
**EMBASE/20,250,115**	**9128**	**10,515**	**10,511**	**4**
**Scopus/20,250,115**	**10,042**	**10,818**	**10,812**	**6**
**Total**	**29,220**	**32,059**	**32,044**	**15**
			**After Dedup.:**	7*

⁎ The sensitivity analysis of the search strategy was conducted on June 17th, 2025. Of the 7 new records identified, 5 were not present in the January 15th, 2025 dataset (Appendix 2A) and they were all irrevant.

**Table:** 

**Search**	**Query**	**Results**
8	5 not 7	**5**
7	(6 or 2) and 3 and 4	**10,721**
6	exp "Sexism"/ or exp "Gender Equity"/ or ("Sexism*" or (("Gender*" or "sex" or "sexual" or "man" or "men" or "Women" or "woman" or "non-binar*" or "male*" or "female*") adj3 ("inequ*" or "prejud*" or "discriminat*" or "bias*" or "stereotyp*" or "exclus*" or "disparit*" or "equit*" or "gap" or "inclus*" or "stigma*" or "parity" or "under-represent*" or "underrepresent*" or imbalanc*))).ti,ab,kf.	68,138
5	(1 or 2) and 3 and 4	**10,726**
4	exp "Intersectional Framework"/ OR exp "Social Theory"/ OR exp "Quality Assurance, Health Care"/ OR ("definit*" or "concept*" or "theor*" or "framework*" or "guideline*" or "polic*").ti,ab,kf.	3409,919
3	exp "Delivery of Health Care"/ or "Professional Practice"/ or exp "Health Care Sector"/ or exp "Right to Health"/ or "Health Policy"/ or "Patient Care"/ or exp "Health Care Quality, Access, and Evaluation"/ or exp "Universal Health Care"/ or ("healthcare" or (("medic*" or "clinic*" or "health*" or "patient*") adj2 ("care" or "service*" or "practice" or "access"))).ti,ab,kf.	10,228,320
2	(exp Health inequities/ or exp Healthcare Disparities/ or Prejudice/ or exp Diversity, Equity, Inclusion/) and (exp Gender identity/ or exp Sex/ or exp Gender role/ or (gender or sex or sexual).ti,ab,kf)	14,208
1	exp "Sexism"/ or exp "Gender Equity"/ or ("Sexism*" or (("Gender*" or "sex" or "sexual" or "man" or "men" or "Women" or "woman" or "non-binar*" or "nonbinar*" or "male*" or "female*") adj3 ("inequ*" or "prejud*" or "discriminat*" or "bias*" or "stereotyp*" or "exclus*" or "disparit*" or "equit*" or "gap" or "inclus*" or "stigma*" or "parity" or "under-represent*" or "underrepresent*" or imbalanc*))).ti,ab,kf.	68,158

**Table:** 

**Search**	**Query**	**Results**
10	#9 NOT #8	**4**
9	#7 NOT ('conference abstract'/it OR 'conference paper'/it OR 'conference review'/it)	**10,515**
8	#6 NOT ('conference abstract'/it OR 'conference paper'/it OR 'conference review'/it)	**10,511**
7	(#5 OR #2) AND #3 AND #4	13,960
6	(#1 OR #2) AND #3 AND #4	13,955
5	'sexism'/exp OR 'gender equity'/exp OR 'sexism*':ti,ab,kw OR ((('gender*' OR 'sex' OR 'sexual' OR 'man' OR 'men' OR 'women' OR 'woman' OR 'non-binar*' OR 'nonbinar*' OR 'male*' OR 'female*') NEAR/3 ('inequ*' OR 'prejud*' OR 'discriminat*' OR 'bias*' OR 'stereotyp*' OR 'exclus*' OR 'disparit*' OR 'equit*' OR 'gap' OR 'inclus*' OR 'stigma*' OR 'parity' OR 'under-represent*' OR 'underrepresent*' OR 'imbalanc*')):ti,ab,kw)	94,998
4	'intersectionality'/exp OR 'sociological theory'/exp OR 'definit*':ti,ab,kw OR 'concept*':ti,ab,kw OR 'theor*':ti,ab,kw OR 'framework*':ti,ab,kw OR 'guideline*':ti,ab,kw OR 'polic*':ti,ab,kw	3909,613
3	'health care delivery'/exp OR 'professional practice'/exp OR 'health care concepts'/exp OR 'right to health'/exp OR 'health care policy'/exp OR 'patient care'/exp OR 'health care quality'/exp OR 'universal health care'/exp OR 'healthcare':ti,ab,kw OR ((('medic*' OR 'clinic*' OR 'health*' OR 'patient*') NEAR/2 ('care' OR 'service*' OR 'practice' OR 'access')):ti,ab,kw)	14,222,543
2	('health disparity'/exp OR 'health care disparity'/exp OR 'prejudice'/exp OR 'diversity, equity and inclusion'/exp) AND ('gender identity'/exp OR 'sex'/exp OR 'sex role'/exp OR 'gender':ti,ab,kw OR 'sex':ti,ab,kw OR 'sexual':ti,ab,kw)	15,562
1	'sexism'/exp OR 'gender equity'/exp OR 'sexism*':ti,ab,kw OR ((('gender*' OR 'sex' OR 'sexual' OR 'man' OR 'men' OR 'women' OR 'woman' OR 'non-binar*' OR 'male*' OR 'female*') NEAR/3 ('inequ*' OR 'prejud*' OR 'discriminat*' OR 'bias*' OR 'stereotyp*' OR 'exclus*' OR 'disparit*' OR 'equit*' OR 'gap' OR 'inclus*' OR 'stigma*' OR 'parity' OR 'under-represent*' OR 'underrepresent*' OR 'imbalanc*')):ti,ab,kw)	94,984

**Table:** 

**Search**	**Query**	**Results**
8	7 not 6	**6**
7	(5 or 2) and 3 and 4	**10,818**
6	(1 or 2) and 3 and 4	**10,812**
5	(TITLE-ABS-KEY ("sexism*" OR (("gender*" OR "sex" OR "sexual" OR "man" OR "men" OR "women" OR "woman" OR "non-binar*" OR "nonbinar*" OR "male*" OR "female*") W/3 ("inequ*" OR "prejud*" OR "discriminat*" OR "bias*" OR "stereotyp*" OR "exclus*" OR "disparit*" OR "equit*" OR "gap" OR "inclus*" OR "stigma*" OR "parity" OR "under-represent*" OR "underrepresent*" OR imbalanc*))) OR AUTHKEY ("sexism*" OR (("gender*" OR "sex" OR "sexual" OR "man" OR "men" OR "women" OR "woman" OR "non-binar*" OR "nonbinar*" OR "male*" OR "female*") W/3 ("inequ*" OR "prejud*" OR "discriminat*" OR "bias*" OR "stereotyp*" OR "exclus*" OR "disparit*" OR "equit*" OR "gap" OR "inclus*" OR "stigma*" OR "parity" OR "under-represent*" OR "underrepresent*" OR imbalanc*))))	199,933
4	(TITLE-ABS-KEY ("definit*" OR "concept*" OR "theor*" OR "framework*" OR "guideline*" OR "polic*") OR AUTHKEY ("definit*" OR "concept*" OR "theor*" OR "framework*" OR "guideline*" OR "polic*"))	16,899,273
3	(TITLE-ABS-KEY ("healthcare" OR (("medic*" OR "clinic*" OR "health*" OR "patient*") W/2 ("care" OR "service*" OR "practice" OR "access"))) OR AUTHKEY ("healthcare" OR (("medic*" OR "clinic*" OR "health*" OR "patient*") W/2 ("care" OR "service*" OR "practice" OR "access"))))	4414,044
2	(TITLE-ABS-KEY ((("health" OR "healthcare") W/4 ("ineq*" OR "dispar*" OR "prejud*" OR "equity" OR "inclusion" OR "diversity")) AND (("gender*" OR "sex" OR "sexual") W/3 ("identit*" OR "role*" OR "orientation"))) OR AUTHKEY ((("health" OR "healthcare") W/4 ("ineq*" OR "dispar*" OR "prejud*" OR "equity" OR "inclusion" OR "diversity")) AND (("gender*" OR "sex" OR "sexual") W/3 ("identit*" OR "role*" OR "orientation"))))	4861
1	(TITLE-ABS-KEY ("sexism*" OR (("gender*" OR "sex" OR "sexual" OR "man" OR "men" OR "women" OR "woman" OR "non-binar*" OR "male*" OR "female*") W/3 ("inequ*" OR "prejud*" OR "discriminat*" OR "bias*" OR "stereotyp*" OR "exclus*" OR "disparit*" OR "equit*" OR "gap" OR "inclus*" OR "stigma*" OR "parity" OR "under-represent*" OR "underrepresent*" OR imbalanc*))) OR AUTHKEY ("sexism*" OR (("gender*" OR "sex" OR "sexual" OR "man" OR "men" OR "women" OR "woman" OR "non-binar*" OR "male*" OR "female*") W/3 ("inequ*" OR "prejud*" OR "discriminat*" OR "bias*" OR "stereotyp*" OR "exclus*" OR "disparit*" OR "equit*" OR "gap" OR "inclus*" OR "stigma*" OR "parity" OR "under-represent*" OR "underrepresent*" OR imbalanc*))))	199,693

## Data Availability

No data was used for the research described in the article.

## Appendix 2a

Search strategy

*Ovid MEDLINE*

***EMBASE***

***Scopus***

## Appendix 2B. Sensitivity analysis comparing search strategies with and without the term “nonbinar*”

**Ovid MEDLINE**

**EMBASE**

**Scopus**

## Appendix 3

• WHO http://www.who.int/en/
• European Institute for Gender Equality (EIGE)

https://eige.europa.eu

• The International society of gender medicine IGM

https://www.intgsm.org/useful-links. This society is linked to:

• The Japanese Association for Gender -Specific Medicine

https://www.jagsm.org/english/index.html

• Gender Basic

http://www.genderbasic.nl

• Gendered innovations in Science, Health & Medicine and Engineering

http://genderedinnovations.stanford.edu

• The foundation for gender-specific medicine

https://gendermed.org

• WHO

https://www.who.int/teams/programme-for-gender-equality–human-rights-and-health-equity

• OGGSM

https://www.gendermedizin.at

• Gendermedizin

https://www.dgesgm.de

• The Israel society for gender and sex conscious medicine

https://www.isragem.org.il/english

• Karolinska Institutet: Renowned for research and education in medical fields, including gender

https://ki.se/en/research/research-areas-centres-and-networks/research-centres/centre-for-gender-medicine

• Centro studi nazionale su salute e medicina di genere

https://www.centrostudinazionalesalutemedicinadigenere.it

• Stanford: the Claymann Institute for Gender Research

https://gender.stanford.edu

• Anna Fischer gender in der medicine

https://gendermed.info

• Juntendo University

https://www.juntendo.ac.jp/english/department/gra/gender_specific_medicine/

**Others relevant sources selected from Tricco et al. 2024** [36]

• CCGSD – Canadian Centre for Gender & Sexual Diversity: http://ccgsd-ccdgs.org
• Centre for Gender Advocacy - Fight gender oppression!: www.genderadvocacy.org
• CIHR Gender and Health Institute: http://www.cihr-irsc.gc.ca/e/8681.html
• European Gender Portal for Equality in Science: http://www.genderportal.eu/
• PAHO: http://www.paho.org/hq/
• Science in Australia Gender equity: http://www.sciencegenderequity.org.au/
• Sexual and Gender Diversity Office (U of T): https://sgdo.utoronto.ca/
• Society for Canadian Women in Science and Technology: http://www.scwist.ca
• U of T Gender Report: http://www.faculty.utoronto.ca/reports/gender-equity-report/genderequity-pathways-to-leadership/
• Institute for Gender and the Economy (GATE) at the Rotman School:

https://www.gendereconomy.org; https://www.gendereconomy.org/research-briefs/

• Gender Summit https://gender-summit.com

**Others suggested by content expert (RT)**

• Global Health 50/50. https://globalhealth5050.org
• Gender Equality, Norms, and Health. Series from the Lancet journals https://www.thelancet.com/series/gender-and-health
• The European Association for Gender Research, Education and Documentation https://atgender.eu
• World Economic Forum. Global Gender Gap Report https://www.weforum.org/reports/global-gender-gap-report
